# Activity-Oriented Antiedema Proprioceptive Therapy (TAPA) for Shoulder Mobility Improvement in Women with Upper Limb Lymphedema Secondary to Breast Cancer: A Multicenter Controlled Clinical Trial

**DOI:** 10.3390/jcm11082234

**Published:** 2022-04-16

**Authors:** María Nieves Muñoz-Alcaraz, Antonio José Jiménez-Vílchez, Mirian Santamaría-Peláez, Luis A. Pérula-de Torres, María Victoria Olmo-Carmona, María Teresa Muñoz-García, Presentación Jorge-Gutiérrez, Jesús Serrano-Merino, Esperanza Romero-Rodríguez, Lorena Rodríguez-Elena, Raquel Refusta-Ainaga, María Pilar Lahoz-Sánchez, Belén Miró-Palacios, Mayra Medrano-Cid, Rosa Magallón-Botaya, Luis A. Mínguez-Mínguez, Josefa González-Santos, Jerónimo J. González-Bernal

**Affiliations:** 1 Inter-Level Clinical Management Unit of Physical Medicine and Rehabilitation, Córdoba and Guadalquivir Health District, Reina Sofía University Hospital, Andalusia Health Service, 14011 Cordoba, Spain; marian.munoz.sspa@juntadeandalucia.es (M.N.M.-A.); maviol@hotmail.es (M.V.O.-C.); mariat.munoz.garcia.sspa@juntadeandalucia.es (M.T.M.-G.); chonjorge@hotmail.com (P.J.-G.); 2 Maimonides Institute for Biomedical Research of Córdoba, Reina Sofía University Hospital, University of Córdoba, 14011 Cordoba, Spain; jesussleep@hotmail.com (J.S.-M.); espe_mrr@hotmail.com (E.R.-R.); 3 Valle de los Pedroches Hospital, Andalusia Health Service, 14400 Pozoblanco, Spain; jimenezvilchez14@gmail.com; 4 Department of Health Sciences, University of Burgos, 09001 Burgos, Spain; mspelaez@ubu.es (M.S.-P.); mjgonzalez@ubu.es (J.G.-S.); jejavier@ubu.es (J.J.G.-B.); 5 Multiprofessional Teaching Unit for Family and Community Care, Córdoba and Guadalquivir Health District, 14011 Cordoba, Spain; 6 San Jose Health Center, Aragonese Health Service, 50013 Zaragoza, Spain; anerol.er@gmail.com (L.R.-E.); minirefus@hotmail.com (R.R.-A.); pilarlahoz95@gmail.com (M.P.L.-S.); 7 Association of People with Lymphedema in Aragon (ADPLA), 50007 Zaragoza, Spain; adplaragon@hotmail.com; 8 Lozano Blesa University Clinical Hospital, Aragonese Health Service, 50013 Zaragoza, Spain; medranocid03@hotmail.com; 9 Institute for Health Research Aragon (IIS Aragon), University of Zaragoza, 50009 Zaragoza, Spain; med000764@gmail.com; 10 Department of Educational Sciences, University of Burgos, 09001 Burgos, Spain

**Keywords:** occupational therapy, breast cancer, secondary lymphedema, upper limb, complex decongestive therapy, multidisciplinary oncological rehabilitation, activity-oriented anti-edema therapy, manual lymphatic drainage

## Abstract

Background: Lymphedema, secondary to breast cancer (BCRL), is the abnormal accumulation of protein-rich fluid in the interstitium caused by a malfunction of the lymphatic system. It causes swelling, deficiencies in upper limb functions and structures, sensory pain and emotional alterations, which have a chronic course and affect the upper limb’s functionality. This study aims to verify the efficacy and efficiency in the upper limb´s functionality of a protocolized experimental approach based on occupational therapy, TAPA (activity-oriented proprioceptive antiedema therapy), in the rehabilitation of BCRL in stages I and II, comparing it with the conservative treatment considered as the standard, complex decongestive therapy (CDT), through a multicenter randomized clinical trial. Methods: a randomized and prospective clinical trial was conducted with experimental and control groups. Women diagnosed with BCRL belonging to institutions in Córdoba and Aragon (Spain) participated. Sociodemographic variables and those related to the functionality of the affected upper limb were evaluated before and after the intervention. Results: The results showed statistically significant differences in the analysis of covariance performed for the variable joint balance of the shoulder´s external rotation (*p* = 0.045) that could be attributed to the intervention performed; however, the effect size was minimal (η^2^ ≤ 0.080). In the rest of the variables, no significant differences were found. Conclusions: TAPA may be an alternative to the conservative treatment of women with BCRL. It was shown to be just as effective for volume reduction and activity performance as CDT but more effective in improving external rotation in shoulder joint balance.

## 1. Introduction

The International Society of Lymphology (ISL) defines lymphedema as an external and/or internal lymphatic system insufficiency manifested by a reduction in general lymphatic transport. It produces swelling due to the accumulation of excess water, diffused filtered plasma proteins, extravascular and parenchymal blood cells and stromal cells products in the extracellular space. The World Health Organization´s International Classification of Diseases includes this disease as a disorder of the circulatory system [1]; its course usually becomes chronic and requires lifelong biopsychosocial treatment, whose results are often not optimal [2].

The most frequent cause of secondary lymphedema in developed countries is secondary to neoplasms, their complications and treatments being the most frequent the upper limb BCRL [3], which is currently the most diagnosed tumor in the world, with more than 2.26 million new cases in 2020 [4].

BCRL can lead to inflammatory complications that cause skin hardening that can break and leave the area exposed to frequent infections, which worsen the BCRL. There may be an alteration of the patient’s sensitivity to touch and kinesthetics, their sexuality perception, self-image problems, decreased levels of physical activity, fatigue, psychological distress, limitations in the strength, endurance and active range of motion of the upper limbs and other sensations related to pain, swelling and heaviness, which decrease the functionality of the affected arm [5,6,7,8,9,10].

There is no clear consensus on the diagnostic criteria for BCRL. The most commonly used system is that of the ISL, based on a three-stage scale for the classification of a lymphedematous limb, from stage 0, subclinical or latent, to stage III, that is lymphostatic elephantiasis [2].

Complex or combined decongestive physical therapy (CDT) is considered the standard in the conservative treatment of lymphedema, with limited evidence [11]. It includes skin care, manual lymphatic drainage, joint mobility exercises, compression garments and multilayer bandages. However, there is no clear optimal treatment strategy, both because of the variability in the protocols, as well as the lack of standardization of results and the variability in the quality of the studies carried out [12]. A wide variety of compression garments are prescribed for lymphedema [13], and the non-serious adverse effects associated with it are identified as skin irritation, discomfort and pain, as well as very rare but serious adverse events, including soft tissue and nerve injuries [14]. It is also contraindicated in arterial insufficiency, severe heart failure and untreated infections [15]. Furthermore, there is no optimal level of adherence to its use because it causes patients to experience discomfort, negative emotions and interference with function and social situations [16]; in addition, its use does not provide a benefit or is contraindicated during the practice of physical activity [17].

Current evidence agrees with the need for further research in multidisciplinary oncology rehabilitation and recommends a multidisciplinary approach in patients with BCRL, including but not limited to occupational therapy [18,19,20,21,22,23,24,25], health training/promotion [9,26,27,28] and physical activity [29,30].

People with BCRL can present alterations of mechanosensitivity [31]. Surgery, chemotherapy and treatment with taxanes can also cause peripheral neuropathies, causing pain and sensitivity alteration in the upper limb, so it is advisable to incorporate neurodynamic activity [32,33]. The literature also recommends adding patterns of proprioceptive neuromuscular facilitation to BCRL rehabilitation [34,35,36]. New cohesive, adaptable bandage systems that provide greater comfort [37,38] are being evaluated, allowing activities and participation in fundamental areas of life.

Based on this evidence, the proposed experimental treatment, TAPA (activity-oriented proprioceptive antiedema therapy), whose results are presented in this article, is compared to the standard treatment, CDT.

The purpose of this study is to evaluate the effectiveness of TAPA as a safe and effective alternative in conservative BCRL treatment to improve the functionality of the upper limb affected by lymphedema, especially in individuals who did not adapt to continuous compression garment use and who had no optimal results.

## 2. Materials and Methods

### 2.1. Study Design

Controlled clinical trial, multicenter, randomized by stratification in two gradients, with two parallel arms and single-blind.

The protocol of this study was published in *BMC Cancer* [39] and was modified from a unicentric to multicenter design in order to facilitate recruitment due to the current state of the SARS-CoV-2 (COVID-19) pandemic, which forced the interruption of activity in the rehabilitation centers, both public and private, of the National Health System. The trial is registered in ClinicalTrials.gov [NCT03762044], dated 23 November 2018: https://clinicaltrials.gov/ct2/show/NCT03762044.

**Figure 1:**
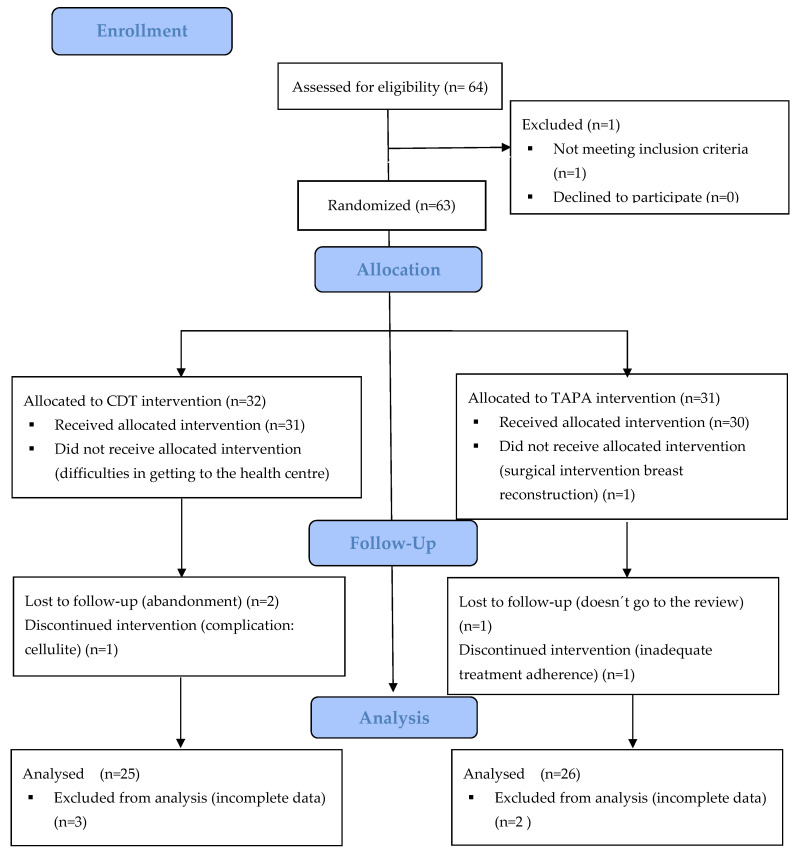
Flowchart. CDT: Complex decongestive physical therapy; TAPA: activity-oriented proprioceptive antiedema therapy.

The study followed the following flowchart (Figure 1):

### 2.2. Study Participants

The study population consisted of women operated on for breast cancer (BC) and diagnosed with upper limb BCRL in stages I and II. They were recruited in two areas: (a) referred to the Clinical Management Unit Inter-levels of Rehabilitation of the Reina Sofía University Hospital of Córdoba and Córdoba and Guadalquivir Health District of the Andalusian Health Service from primary and specialized care and (b) derived from the Association of Aragonese Women with Genital and Breast Cancer (AMAC-GEMA) and Association of People with Lymphedema of Aragon (ADPLA) to the San José Health Centre and University Clinical Hospital of the Aragonese Health Service.

Inclusion criteria were women operated on for BC with BCRL in stages I and II (as defined in the consensus document of the International Society of Lymphology 2020 [2]) and those who signed the informed consent form. The study excluded patients with health problems, diseases or dysfunctions that prevented them from participating in the intervention or those with bilateral lymphedema.

It was determined that a reduction of 150 mL in the volume of lymphedema could be established as relevant (minimum detectable value), approximately 20% with respect to baseline [40]. Taking into account the data from the literature on means and standard deviations obtained in other studies [35,41], for an alpha error of 0.05 and a statistical power of 80%, the necessary sample size would be 29 subjects per group (calculated with EPIDAT 4.2). It was assumed that the effect of the standard treatment in the control group (CG) would result in a reduction in arm volume by an average of 5%, the treatment effect in the experimental group (EG) would cause an average reduction in arm volume of 20% and the standard deviation would be similar in both groups, approaching 20% [42]. Considering a dropout rate of 10% in each group, the estimated corrected sample size was 64 patients, randomly assigned to two groups of 32 patients each, with 16 per stage for each treatment group (CG: 16 in stage I and 16 in stage II. EC: 16 in stage I and 16 in stage II).

### 2.3. Procedure and Randomization

Consecutive sampling was performed in which patients who met the criteria were invited to participate in the study as they were identified and recruited. The patients were grouped by stratified random selection using the statistical software EPIDAT, 3.1, which stratifies patients by lymphedema stage in a 1:1 ratio using random-sized blocks of four. The assignment sequence was concealed from the evaluating researcher using numbered, opaque, sealed and stapled envelopes. The researchers in charge of monitoring and analyzing the data (a Specialist in Preventive Medicine and Public Health, a nurse and a Specialist in Family and Community Medicine) were also blinded to the result after the interventions were assigned.

The degree of interobserver reliability between the different evaluators in the measurement of the circometry of both arms (affection and healthy) in 4 volunteer subjects was analyzed, with 7 measurements in 7 predefined anatomical regions and with 5 cm of difference between them in each of them. The interobserver agreement for these measurements was analyzed by measuring the intraclass correlation coefficient (ICC) with Epidat 4.2 software, obtaining a value of 0.60, which indicates a good degree of agreement. For the interpretation of the CCI, the classification obtained by Fleiss was used [43], according to which a CCI > 0.91 corresponded to very good concordance; 0.71–0.90, good; 0.51–0.70, moderate; 0.31–0.50, mediocre and if it was <0.30, the agreement would be bad or very bad.

### 2.4. Main Outcomes

The degree of lymphedema was measured by calculating the volume difference between the affected upper limb and the contralateral limb as a percentage and in millilitres (mL). According to the percentage of volumetry through circometry (manual measurement of limb perimeters with measuring tape, the volume value is approximate and volume calculation is according to Kuhnke’s formula), Vol = (C1 2 + C2 2 + … Cn 2)/π) [44,45,46]. A distinction was made between Grade 1 or mild (difference in volume 5–20% with respect to the healthy arm) and Grade 2 or moderate (difference in volume 20–40% with respect to the healthy arm). According to volumetry expressing the volume in mL by the difference in volume as a % between both extremities, a reduction of edema of 150 mL (20%) over baseline was required to be considered clinically relevant [47].

Shoulder joint balance for flexion (JBF), abduction (JBABD) and external rotation (JBER) was measured with a goniometer.

Upper limb function/activity performance: Measured with the Quick Disabilities of the Arm, Shoulder and Hand (Quick-DASH), with cross-cultural adaptation, reliability, validity and sensitivity to changes from its extended version in 2006 [48,49,50]. For the value of the Quick-DASH, the result was calculated as a percentage; the higher the result obtained, the greater the disability or symptom.

### 2.5. Statistical Analysis

Statistical analysis was performed with the intention to treat. Quantitative variables are described as the mean, standard deviation and limits of each distribution and qualitative variable as absolute and relative frequencies. A bivariate analysis was performed, verifying that the quantitative variables follow a normal distribution, using the Shapiro–Wilk test. After that, the differential scores for all the continuous variables were calculated by subtracting the pretest score from the post-test score; these differential scores were used in the covariance analysis (ANCOVA) to check if statistically significant differences appeared between CG and EG for the variables analyzed. The pretest score of the dependent variable was used as a covariate and the intervention group was used as a fixed factor. The effect size was estimated using the eta square coefficient (η^2^) so that if 0 ≤ η^2^ < 0.05, there was no effect; if 0.05 ≤ η^2^ < 0.26, the effect was minimal; if 0.26 ≤ η^2^ < 0.64, the effect was moderate; and if η^2^ ≥ 0.64, the effect was strong [51]. The statistical analysis was performed with the statistical package for social sciences (SPSS v.28), establishing a statistical significance value of *p* < 0.05.

### 2.6. Intervention

Both groups received a 3 h health education workshop on lymphedema prior to the intervention, in which basic knowledge about the pathophysiology of lymphedema, early identification of symptoms, preventive skin care measures, guidance for ADL performance, including physical activity, and recommendations for exercises and anti-edema postures to be performed at least once a day were taught.

The EG group applied the TAPA treatment to stages I and II. They received 10 sessions (2 weekly) of 30 min each, led by two occupational therapists, one from the health district of Córdoba and Guadalquivir and another from the San José de Zaragoza Health Centre. This treatment consisted of myolinfokinetic therapeutic activity which reduced the volume of lymphedema and was significant for each individual. It also involved the measurement and graduation of neurodynamic components, the proprioceptive neuromuscular facilitation of significant activities and a proprioceptive cohesive anti-edema bandage, similar to a Coban type bandage, single layer, without compression and with high cotton content. The patient and/or caregiver was instructed on its use and placement, and modifications/adaptations were recommended for optimal performance in their activities of daily living (ADL). After the 10 sessions, each patient had to perform 5 individually prescribed daily activities and was told not to wear any compression garment.

The intervention of the CG in stage I was developed by a Physical Medicine and Rehabilitation Specialist and a Family and Community Medicine Specialist, and in stage II by two physiotherapists, one from the UGC Interleaved of Physical Medicine and rehabilitation and the other from the University Clinical Hospital of Zaragoza and consisted of action collected by the Integrated Breast Cancer Care Process of the Ministry of Health [52]. Stage I consisted of preventive measures, skin care, exercise and the use of compression garments (duration of 5 weeks). Stage II consisted of 10 60 min sessions with conservative CDTY treatment, three times per week, as usual, with the full CDT session length, but there are no protocols that defined specific guidelines for the application of the treatment, leaving it to the professional´s discretion according to the patient´s state. Stage II patients also received skin protection, multilayer bandages, manual lymphatic drainage and were told to wear a compression garment.

The project was approved by the Research Ethics Committee of Córdoba in a meeting held on November 27, 2018 (Act no. 282, ref. 4084) and the authorization of the Management/Direction of the Health District of Córdoba and Guadalquivir, the Reina Sofía University Hospital and the AECC Headquarters Córdoba.

The principles established in the Declaration of Helsinki of 1964, of the World Medical Association and subsequent amendments and the 1996 Council of Europe Convention on Human Rights and Biomedicine, as well as the requirements established in Spanish legislation, were respected. The investigation complied with the rules of good clinical practice (art. 34 RD 223/2004; community directive 2001/20/EC), the protection of personal data and confidentiality (European Data Protection Regulation, and in accordance with Organic Law 3/2018 on the Protection of Personal Data and guarantee of digital rights). In the development of the study, Law 41/2002 on Patient Autonomy and Law 14/2007 on Biomedical Research were considered.

## 3. Results

### 3.1. Main Characteristics of the Participants

Of the 63 women who were recruited for this study, 51 finished it, 25 belonged to the GC and 26 to the GE. There were 12 losses as a result of inadequate adherence to treatment and data collection issues.

The mean age was 59.24 years (SD ± 9.55), and they were mainly active (*n* = 26; 51%) or retired (*n* = 19; 37%). In relation to the surgical intervention, two participants (4%) underwent breast-conserving surgery, while the rest underwent a mastectomy (*n* = 49; 96%). In most cases (*n* = 43; 84%) there were no post-surgical complications.

### 3.2. Volume, Joint Balance and Upper Limb Function: Differences between Groups

Table 1 show the descriptive results of the study, comparing them basally according to the group. Only a statistically significant difference in age was observed (*p* = 0.026).

The data in Table 2 show the main results obtained from the Covariance Analysis (ANCOVA) performed for each of the continuous variables in order to determine if there were significant statistical differences between the GC and EG after the intervention.

ANCOVA showed statistically significant differences between GC and GE in the joint balance of the external rotation of the shoulder, controlling the scores obtained in the pretest, which could be attributed to the intervention performed. This means that the JBER scored higher at the end of the EG and improved more in the group of participants who received the TAPA treatment based on activity as a treatment method and without compression in the upper limb of the affected side, compared to the group that received the conventional treatment. Despite being significant, the effect size was (η^2^ ≤ 0.080), and no statistically significant differences were obtained in the other dimensions of functionality and volume studied between the two intervention groups. However, there were very significant differences (*p* < 0.001) in the percentage of edema volume reduction and in the shoulder flexion joint balance improvement (*p* = 0.013) within patients who underwent the experimental intervention with respect to their baseline situation, although this was not correlated with statistically significant improvements between groups for the performance of upper limb activities evaluated with QuickDash (*p* = 0.464).

## 4. Discussion

The results of this study do not show significant differences in the volume reduction of BCRL between the intervention groups (control and experimental) that are consistentwith the review performed by Jeanette Ezzo et al. [53]. However, the decrease in volume reduction with the experimental treatment, TAPA, was very significant with respect to the baseline evaluation of the patients. On the other hand, they observe contradictory results in the function of the range of motion, while this research shows a significant improvement in the joint balance of external shoulder rotation. The experimental intervention also improved significantly the shoulder flexion joint balance.

In this line of results, the meta-analysis by Flávia Belavenuto Rangon BS et al. [54] notes, like our findings, found that there were no statistically significant differences in the effect between CDT and short-term multimodal approaches on volume reduction. These authors also found no significant differences regarding upper limb physical function when compared with TAPA.

Paolo Marchica et al. [55] reviewed the state of the art treatment of BCRL, noting that only bandages and intermittent pneumatic pressure showed a substantial beneficial effect in reducing lymphedema volume in the acute-intensive phase and that manual lymphatic drainage, an essential component of CDT, was not effective for BCRL. They also pointed out that physical activity remains a milestone in BCRL as it reduces volume and improves upper limb strength; these conclusions support TAPA’s effectiveness since it uses significant activity and a proprioceptive bandage as treatment methods, with optimal results both in volume reduction and upper limb function. TAPA facilitates therapeutic decision-making, establishing treatment doses, as it involves an intervention protocol with a specific number of sessions and activities and with a treatment duration also defined, with five activities and a self-bandage that is not compressive but proprioceptive. Currently, the number of hours and contexts in which the patient needs to use the compression garments is left to the therapist’s discretion, as well as the frequency and need for rehabilitative treatment and its temporalization.

One of the strengths of this study is that it stands as an effective therapeutic alternative for people with BCRL who have contraindications to perform CDT, as well as for those who do not wish to use compression garments while eliminating the possible adverse effects of different compressive treatments.

Another important aspect of this study is that it can help to plan the care and needs of people with BC by increasing, to a greater degree, the joint balance of the external rotation of the shoulder compared to conventional treatments since, as observed by Roser Belmonte et al. [56], the loss of strength for external rotators and range of motion of the shoulder and health-related quality of life in the physical domains and the arm persists at 5 years in the groups of patients operated on for BC. Similarly, Emine Baran et al. [57] described that BCRL patients have a lower active shoulder range of motion on all measures than unaffected people without BCRL.

The authors consider it a limitation of the study not to have evaluated the joint balance of the elbow since it could have provided additional information of interest on the impact of the loss of joint range in the difficulties of carrying out activities and problems of restriction of participation as shoulder and elbow angles are necessary to perform activities of daily living. Still, elbow angles are considered of greater relevance [58], which could justify that significant improvements in upper limb functionality measured with QDASH were not found in this randomized clinical trial.

This research provides information on the effect of each intervention depending on lymphedema stages, but not on other relevant variables such as types of treatment or surgeries; current evidence identifies clear risk factors for lymphedema secondary to breast cancer axillary node dissection and regional radiation of axillary nodes. In addition, conservative surgery through sentinel node biopsy has shown a promising reduction in postoperative node incidence [59]. It is also considered that further research would be necessary to describe the effects of the experimental treatment in all stages of BCRL, as well as in BCRL in men, to continue the advance in informed therapeutic decisions.

## 5. Conclusions

Activity-Oriented Proprioceptive Antiedema Therapy (TAPA) may be an alternative in the conservative treatment of women with BCRL. It is just as effective in reducing volume and performing activities as CDT but it is more effective in improving external rotation in shoulder joint balance.

TAPA is an effective treatment, simpler in terms of organization, which is something to consider for the optimization of resources.

## Figures and Tables

**Table 1:** Analysis of the baseline comparability of both results.

Variable	Type of Treatment Received	*n*	Mean	SD	SEM
Age	Experimental	31	58.00	9.71	1.74
	Control	32	63.66	9.96	1.76
Body mass index	Experimental	30	28.76	4.73	0.86
	Control	31	27.59	4.91	0.88
Number of nodes removed	Experimental	29	14.00	9.73	1.80
	Control	24	12.20	7.21	1.47
Pain, VAS scale 0–10	Experimental	31	3.77	2.82	0.50
	Control	32	3.88	3.11	0.55
Heaviness, VAS scale 0–10	Experimental	31	4.87	2.79	0.50
	Control	32	4.88	2.25	0.39
Tightness, VAS scale 0–10	Experimental	31	4.19	2.67	0.48
	Control	32	4.38	2.72	0.48
Health Questionnaire	Experimental	31	67.10	18.42	3.31
	Control	30	64.83	16.47	3.00

SD: Standard deviation; SEM: Standard error mean.

**Table 2:** Comparison between groups in continuous variables differential punctuation, controlling pretest scores. ANCOVA.

Variable	Source	Type III Sum of Square	df	MS	F	*p*-Value	η^2^
Differential Vol%	Vol% pre-test	194,568,957	1	194,568,957	1,809,568	<0.001	0.974
	CG/EG	82,518	1	82,518	0.767	0.385	0.016
	Error	5,161,072	48	107,522			
Differential Vol mL	Vol mL pre-test	8,698,016	1	8,698,016	0.478	0.493	0.010
	CG/EG	10,305,528	1	10,305,528	0.567	0.455	0.012
	Error	872,983,058	48	18,187,147			
Differential QDASH	QDASH pre-test	3325	1	3.325	0.019	0.890	0.000
	CG/EG	94,477	1	94.477	0.545	0.464	0.011
	Error	8,317,476	48	173.281			
Differential JBER	JBER pre-test	200,036	1	200,036	6289	0.016	0.114
	CG/EG	135,014	1	135,014	4245	0.045	0.080
	Error	1,558,617	49	31,809			
Differential JBF	JBF pre-test	624,712	1	624,712	6723	0.013	0.121
	CG/EG	128,549	1	128,549	1383	0.245	0.027
	Error	4,553,173	49	92,922			
Differential JBABD	JBABD pre-test	239,799	1	239,799	1171	0.284	0.023
	CG/EG	477,384	1	477,384	2331	0.133	0.045
	Error	10,034,239	49	204,780			

Vol%: degree of lymphedema according to volumetric percentage; Vol mL: degree of lymphedema according to the volume expressed in mL; QDASH: upper limb function; JBER: joint balance external rotation; JBF: joint balance flexion; JBABD: joint balance abduction; MS: mean square; CG: Control Group; EG: Experimental Group.

## Data Availability

Not applicable.
